# Patterns of distribution, population genetics and ecological requirements of field-occurring resistant and susceptible *Pseudosuccinea columella* snails to *Fasciola hepatica* in Cuba

**DOI:** 10.1038/s41598-019-50894-7

**Published:** 2019-10-07

**Authors:** Annia Alba, Antonio A. Vázquez, Jorge Sánchez, Manon Lounnas, Jean-Pierre Pointier, Sylvie Hurtrez-Boussès, Benjamin Gourbal

**Affiliations:** 1 Centro de Investigaciones, Diagnóstico y Referencia, Instituto de Medicina Tropical “Pedro Kourí”, Apartado Postal 601, Marianao 13, 11400 La Habana, Cuba; 2 IHPE, Univ. Montpellier, CNRS, Ifremer, Univ. Perpignan Via Domitia, Perpignan, France; 3 Mivegec UMR UM, CNRS 5290 – IRD 224 Maladies Infectieuses et Vecteurs: Ecologie, Génétique, Evolution et Contrôle, Centre IRD, BP 64501, 34394 Montpellier, Cedex 5 France; 4 PSL Université Paris: EPHE- UPVD-CNRS, USR 3278 CRIOBE, Université de Perpignan, 52 Avenue Paul Alduy, 66860 Perpignan, Cedex France; 5 Département de Biologie-Ecologie (Faculté des Sciences) -cc 046- Université Montpellier, 4 Place Eugène Bataillon, 34095 Montpellier, Cedex 5 France

**Keywords:** Ecology, Genetics

## Abstract

*Pseudosuccinea columella* snails transmit the trematode *Fasciola hepatica*, but in Cuba, six naturally occurring populations successfully resist parasite infection. Here, we present an updated distribution of *P*. *columella* in Cuba; 68 positive sites with the earliest records more abundant in west-central Cuba and with east-central populations generally corresponding to the newest samples. No records were found farther east. The IPA site reported 10.5% prevalence of *F*. *hepatica-*infected snails. Population genetics, studied through microsatellites, showed low allelic and multilocus genotypic richness (MLGT), mainly in susceptible populations, strong deviations from panmixia and high self-fertilization rates. Susceptible individuals were grouped in one major cluster containing the majority of MLGT, and two independent clusters grouped the MLGT of resistant individuals from western and central populations, respectively. From these, we propose that several introductions of *P*. *columella* occurred in Cuba, primarily in the west, with the early arrivals deriving on the resistant populations. A more recent introduction of susceptible *P*. *columella* carrying MLGT *T* and *Y* may have occurred, where the latter spread quickly through the island and possibly increase the risk of parasite transmission in Cuba since all snails naturally infected with *F*. *hepatica* were carriers of the MLGT *Y*. Interestingly, even though resistant populations are highly diverse and are likely the oldest within Cuba, they are only found in six localities characterized by soft (total hardness, TH = 6.3 ± 1.03°d) and slightly acidic (pH = 6.2 ± 0.12) waters with low richness in snail species (3.2 ± 1.02). This tendency was also observed in a two-year follow-up ecological study that was conducted on a farm where both phenotypes occurred in sympatry; colonization events by resistant over susceptible snails coincided with a reduction in the pH and TH of the water. A comparison of life traits in susceptible and resistant isolates reared at two different pH/TH conditions (5.9/4°d or 7.8/14°d) showed that low pH/TH negatively affects *P*. *columella*, irrespective of the phenotype. However, evidence of higher tolerance (higher survival, life expectancy, egg viability) to such conditions was observed in resistant isolates. Finally, we speculate that the limited distribution of resistant populations might be related to a better exploitation of sites that are less suitable to snails (thus, with lower competition), rather than to a differential ecological restriction to specific environmental conditions from susceptible *P*. *columella*.

## Introduction

Freshwater snails of the family Lymnaeidae (also known as pond snails) inhabit tropical, temperate and cold regions, from sea level to high altitudes^1^. Many of the nearly 100 species recently attributed to this family^2^ have become the target of taxonomical and parasitological studies over the past few years, due to their important roles as intermediate hosts of several medically relevant parasites, including the globally distributed liver fluke *Fasciola hepatica* (*e*.*g*.^3,4^). Within Lymnaeidae, several species have been incidentally carried out of their native range and introduced elsewhere (see the cases of *Galba cubensis* and *Austropeplea viridis* in Europe;^5,6^). One of the most interesting examples is that of *Pseudosuccinea columella* (Say, 1817), a globally introduced species with a considerable invasive ability^7^. The transmission of *F*. *hepatica* by *P*. *columella* is well-documented in its native range (North America; see^8^) and in places where it has been introduced such as Brazil^9^, Argentina^10^ and Australia^11^. This species is also thought to enhance transmission in remote regions where it has invaded, like in Egypt^12^ and the Pacific islands, including Hawaii and French Polynesia^13,14^.

In Cuba, the first report of *P*. *columella* dates back to 1858 in the western region and the latest malacological surveys depict a western-central distribution^15^. Although this species is thought to play a secondary role in *F*. *hepatica* transmission in Cuba (with *Galba cubensis* being the main host; see^16,17^), it is found naturally infected in the field^18^. Important investigations have been carried out on *P*. *columella* in recent decades due to the existence of a resistant phenotype against *F*. *hepatica* infection in certain natural Cuban populations^19,20^. Experimental exposures of these resistant snails to different sympatric and allopatric fluke isolates always result in the failure of parasite larval stage development^19–22^ which is associated with an effective encapsulation of the parasite by snail immune cells^19^.

Interestingly, thus far, all resistant *P*. *columella* can be easily differentiated from susceptible individuals in the field though the use of a phenotypic marker consisting of a belt-like pattern of small, sharp and whitish spots in the mid-region of the mantle with bigger spots scattered on the upper and lower sides^19^. Attempts to genetically differentiate susceptible from resistant individuals have independently clustered both phenotypes by RAPD markers^21^. More recently, Cuban resistant populations were segregated from *P*. *columella* populations found elsewhere, using mitochondrial haplotypes and microsatellites markers^7^. It is important to stress that the observed molecular and phenotypic differences are not a reliable mean of separating resistant populations as a different species. Resistant snails displayed the same reliable features of the shell and the same reproductive system anatomy defined for *P*. *columella* with only a slight difference in two-nucleotide changes (0.17%) within the ITS1 and ITS2 when ribosomal genes were sequenced and compared^23^. From an ecological point of view, previous comparative studies on demographic dynamics in the laboratory between susceptible and resistant snails have suggested the existence of a trade-offs against reproduction of the latter^24,25^.

The natural occurrence of populations that are susceptible and resistant to a parasite within the same host species provides a unique model that can be addressed from several disciplines, including evolutionary biology and host-pathogen interaction, environmental and health sciences and snail control. Here, for the first time, we present a global overview of the ecology of *P*. *columella* in Cuba, where both phenotypes are considered separately by the use of different approaches. We present its updated distribution, study its infection status and its genetic structure by microsatellites markers, and elucidate the ecological patterns associated with the occurrence of resistant and susceptible populations through the analysis of field data and experimental life history traits. From these, we gain insight into the history, colonization and population structure of *P*. *columella* in Cuba, its relationship with *F*. *hepatica* and the increasing risk of parasite transmission represented by certain widespread genotypes. In addition, we provide further knowledge related to habitat preferences of *P*. *columella* and the environmental conditions that contribute to its occurrence in the wild. All of these aspects are essential to understand and to predict the distribution pattern and further spreading of *P*. *columella* snails in Cuba. Findings from this study may play an important role in conservation biology, parasitology and epidemiology and to devise intermediate host control strategies based on the potential application of *P*. *columella* resistance.

## Material and Methods

### Distribution of *P*. *columella* in Cuba

The updated distribution of *P*. *columella* was obtained from the National Reference Malacological Database for freshwater snails in Cuba, held by the Laboratory of Malacology of the Institute of Tropical Medicine in Havana, through the revision of 496 sampled sites ( = populations) from 1982 to 2018. Each record includes the presence of a given freshwater snail, geographic coordinates, habitat type, date and collector. Nationwide malacological surveys are carried out by our laboratory on a 10-year sampling period basis complemented with annual expeditions covering smaller areas. All sites positive for *P*. *columella* were plotted on a map differentiating each record according to a 11-year time interval, to account for new and historical data, using MapInfo v.15 (Pitney Bowes Software Inc., New York, USA, 2015). The frequency of each habitat type where *P*. *columella* was observed was used as a measure of habitat preference.

Differentiation of resistant and susceptible *P*. *columella* populations to *F*. *hepatica* infection were primarily based on the presence or absence of a characteristic band of small sharp spots in the mid-region of the mantle, as its occurrence is associated with the resistant phenotype^19^. Additionally, both phenotypes, and particularly that of the resistant populations, were double-checked in the laboratory by experimental infection using different panels of potentially sympatric (retrieved from infected cattle reared at the site or in nearby areas) and allopatric *F*. *hepatica* isolates (e.g.^20–22^). The latter confirmed the reliability of the mantle pigmentation pattern as a distinctive phenotypic marker for *F*. *hepatica* resistance in *P*. *columella*. Experimental infections were carried out following the methodology previously described^20^, exposing 30 snails per population to a dose of five *F*. *hepatica* miracidia.

### Population genetics of *P*. *columella* from Cuba

#### Snail sampling, DNA extraction, amplification and genotyping of microsatellite loci

We sampled 20 populations of *P*. *columella* (17 susceptible and 3 resistant, *i*.*e*. displaying the reliable phenotypic mantle marker described by Gutiérrez *et al*.^19^). When possible, up to 30 individuals were collected from each population, up to a total of 329 snails. The snails were carefully dissected in the laboratory for screening of possible parasite infection. A piece of tissue from the foot was used for DNA extraction to avoid foreign genetic contamination (*e*.*g*. sperm from cross-fertilization). The extraction of DNA was performed using Chelex methodology following^26^ and slightly modified for 96-well plates. In brief, a small portion of tissue was added to a mixture of 100 µL of 5% Chelex®100 (Bio-Rad) and 5 µL of proteinase K (50 mg/mL; Promega) in each well. The plate was mixed in vortex, incubated overnight at 56 °C and placed for 10 min at 95 °C. The mixture was centrifuged at 6000 × *g* for 6 min and the supernatant containing the DNA was collected and stored at −20 °C until use.

PCR amplifications were performed in a 96-well MJ-Research PTC 100 for all samples. Six microsatellite loci, previously described for *P*. *columella* species by Nicot *et al*.^27^, were amplified (GenBank Access number): PCO01 (EU04295), PCO02 (EU049296), PCO07 (EU049299), PCO12 (EU049303), PCO13 (EU049304) and PCO20 (EU049309). Each locus was amplified through PCR using 1 µL of extracted DNA in 10 µL of reaction volume containing 2 µL buffer 5 × (Promega), 1 µL of 25 mmol/L MgCl_2_, 0.5 µL of 2 mmol/L dNTP (Invitro-gen/Life Technology), 0.2 µL of each primer (10 pmol) and 0.2 µL of *Taq* DNA polymerase (Promega). Thermocycling consisted of 10 min of initial denaturalization at 94 °C, 30 cycles at 94 °C for 30 s, annealing at 55 °C (for each locus) for 30 s, 60 s at 72 °C and a final elongation step at 60 °C for 30 min.

Primers were fluorescently labelled to be used in an ABI automated sequencer (ABI Prism 310 Genetic Analyser, Applied Biosystem, Perkin-Elmer, USA). Amplified products for each individual were used for sequencing where 1 µL of diluted amplicon (1/100) was added to a mix containing 15 µL of Hi-Di Formamide and 0.27 µL of internal size standards (GENESCAN 500 LIZ, Applera). Each allele’s length was read using GeneMapper® v.4.0 software (Applied Biosystems^TM^).

#### Population genetics analysis

Current parameters of population genetics like the mean number of alleles (*a*), the observed (*H*_o_) and expected (*H*_s_) heterozygosity and *F*_IS_ were estimated for each locus. Pairwise differentiation between populations (*F*_ST_) was also tested. Calculations were computed using FSTAT v2.9.3.2^28^ and Bonferroni corrections were applied for multiple tests^29^. The selfing rate (*s*) for each population was calculated using the equation *s* = 2*F*_IS_/(1 + *F*_IS_)^30^. Identical multilocus genotypes (MLGT) were identified based only on individuals with all microsatellite loci amplified and coded using a combination of letters (one letter or letters’ combination = one MLGT = unique combination of alleles across all analysed loci). Nei’s^31,32^ genetic distances between populations were estimated using Genetix^33^. The obtained distance matrix was used to build a neighbour-joining network of all MLGT in SplitsTree4^34^. The genotypic diversity was calculated in each population with an adaptation of the Simpson index using GENCLONE 2.0^35^.

### Ecological patterns of resistant and susceptible *P*. *columella*

The ecological patterns of the two phenotypes of *P*. *columella* were explored using three approaches: laboratory field records, field study and experimental life tables.

#### Revisiting laboratory field records of resistant and susceptible P. columella populations

A thorough literature review which included published papers and 37 years of unpublished data from our laboratory’s field notes, was conducted. We gathered data for species composition of the mollusc assemblage and field abiotic factors (phosphates, nitrates, dissolved oxygen, pH and total hardness) measured for resistant and susceptible populations investigated within this study.

#### Ecological follow up of resistant and susceptible populations of P. columella

**Figure 1:**
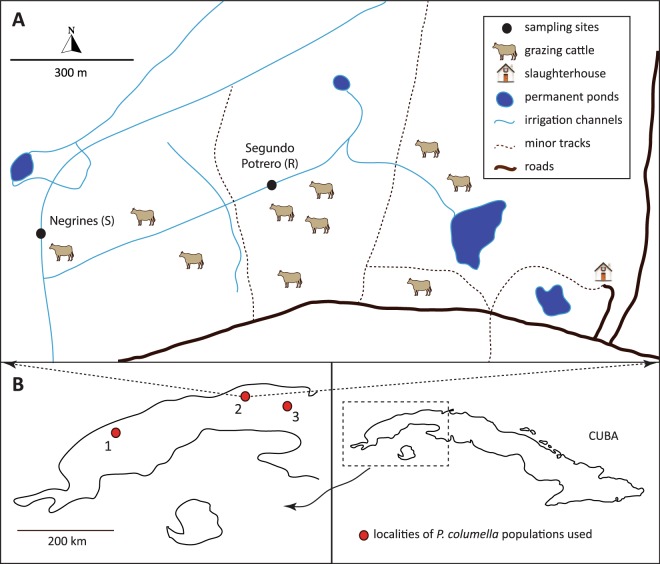
(**A**) Overview of the locality La Coca showing the two sites, *i*.*e*. Segundo Potrero and Negrines, sampled during the two-year follow-up ecological study where the resistant (R) La Coca population and the susceptible (S) Negrines population of *Pseudosuccinea columella* occurred. (**B**) Localities from western Cuba where the four populations of resistant (1: La Palma, 2: La Coca) and susceptible (2: Negrines within La Coca locality, 3: Aurora) *P*. *columella* used in the life table experiments were collected from.

We performed a two-year follow-up ecological study on the La Coca (=locality La Coca) cattle farm located in south-western Havana, as it harbours resistant and susceptible populations of *P*. *columella* (see Fig. 1A). The region is characterized by a lowland meadow that is commonly flooded during the rainy season, which extends for nearly 10 km^2^. The area includes several channels used for irrigation and livestock watering, usually no more than 60 centimetres in depth. Gramineous vegetation is abundant but aquatic plants in the channels are scarce. These features contribute to the presence of several water bodies (sites) scattered throughout the locality where freshwater snail populations thrive. The *P*. *columella* species is present at five sites from the La Coca locality, four of which harbour susceptible populations. The fifth site is known as Segundo Potrero and is characterized by the occurrence of a resistant *P*. *columella* population named La Coca after the locality.

Thus, based on these characteristics, we selected two sites from the locality La Coca for the ecological follow-up: (i) Segundo Potrero, due to the presence of the resistant *P*. *columella* population (La Coca population) and (ii) a nearby site called Negrines, 500 m away and connected by one of the channels, where a population of susceptible individuals (Negrines population) occurs. Each site was sampled at monthly intervals from January 2011 to December 2012 always by the same person during the early hours of the morning, using soft forceps and sieves (1 mm mesh). We used the capture per unit of effort method over a 15 minute interval^36^ and relative abundance was measured as individuals/15′ for each species. Aquatic and shore vegetation were swept for every sample. Resistant snails were easily identified in the field by the phenotypic marker of mantle pigmentation^19^. Abiotic factors including pH, temperature, total hardness (TH), carbonate hardness (CH), dissolved oxygen, phosphates and nitrites were measured using a field kit (Merck). Species diversity was calculated for each site using the Simpson index^37^ and a canonical correspondence analysis^38^ was done to detect unimodal response of mollusc species abundances to ecological factors.

Additionally, all collected lymnaeid snails were brought to the laboratory and dissected to account for *F*. *hepatica* larval stage infection.

#### Laboratory life tables of resistant and susceptible P. columella

Four natural populations of *P*. *columella*; two composed of susceptible (Negrines, 22.9577°N; −82.4649°W and Aurora, 23.0789°N; −81.9176°W) and two of resistant (La Coca, 22.9554°N; −82.4598°W and La Palma, 22.7695°N; −83.5443°W) individuals (see Fig. 1B), were sampled and reared under laboratory conditions for one generation. Given the results from field data concerning the ecological patterns related to the occurrence of both phenotypes, two different conditions of pH/total hardness (TH) were set up: common (7.6/14°d; according to Perera^36^, those conditions are the most commonly found for Cuban freshwater habitats) and lower (5.9/14°d) conditions. Snails were then reared in the two conditions using separate 25 L aquariums. Five-day-old snails from each isolate (n = 30 per population at each condition) were placed in the aquariums and this moment of transfer was considered as time zero for the experiment. All aquariums were provided with 50 mg each of CaCO_3_ and MgCO_3_ to contribute to shell formation. Water acidification down to pH 5.9 (low pH condition) was achieved through an automatic CO_2_ pump connected to a setter pH meter. Total hardness conditions were adjusted to the required range for each aquarium by differentially mixing aged tap water with distilled water. Both pH and TH were systematically checked along with other abiotic parameters (Cl^−^, NO_3_^−^, NO_2_^−^, CH) with a commercial aquarium kit to guarantee optimal and reproducible conditions during the experiment. Temperature was always maintained at 25–26 °C and the snails were fed three times per week with a mixture of algae cultured for this purpose following Sánchez *et al*.^39^. All populations were followed until the death of all individuals. Live and dead snails, the number of eggs, egg masses and newly hatched snails (NHS) were counted at weekly intervals. At the end of the experiment, the following life table parameters were determined according to Margalef^40^: proportion of living individuals (lx), probability of survival (px), life expectancy (ex), fecundity rate (mx), net reproduction rate (Ro), and intrinsic (r) and finite (λ) rates of natural increase.

Snail survival curves were constructed based on the percentage of live snails at each time point and pairwise log-rank tests of Kaplan-Meier curves were performed to assess the statistical differences on survivorship data. The factorial ANOVAs followed by the *post hoc* multiple comparison Tukey test were carried out to assess statistical significance between the mean values of eggs, egg masses and egg/egg masses per population. The overall percentage of viable eggs (*i*.*e*. viable eggs = NHS/number of eggs) per strain at each condition was compared through a Fisher’s Exact Test. Similarly, the percentage of NHS from the first, second and third week after egg laying was determined for each strain and compared between experimental settings to assess possible delays. Statistica v.12 (StatSoft. Inc., Tulsa, OK, USA 2014) software was used for calculations and differences were considered significant when *P < *0.05.

### Ethics approval

French laboratory holds permit #A66040 for experiments on animals, which was obtained from the French Ministry of Agriculture and Fisheries and the French Ministry of National Education, Research and Technology. The housing, breeding and care of the utilized animals followed the ethical requirements of France. The experimenter possesses an official certificate for animal experimentation from both of the above-listed French ministries (Decree #87–848, October 19, 1987). The various protocols used in this study have been approved by the French Veterinary Agency of the DRAAF Languedoc-Roussillon (Direction Régionale de l′Alimentation, de l′Agriculture et de la Forêt), Montpellier, France (authorization # 007083). The Cuban laboratory of Malacology holds permit for experiments on animals obtained from the Cuban government. The housing, breeding and care of the utilized animals followed the ethical requirements of Cuba. The experimenter possesses an official certificate for animal experimentation from the Cuban government.

## Results

### Distribution of *P*. *columella* in Cuba

**Figure 2:**
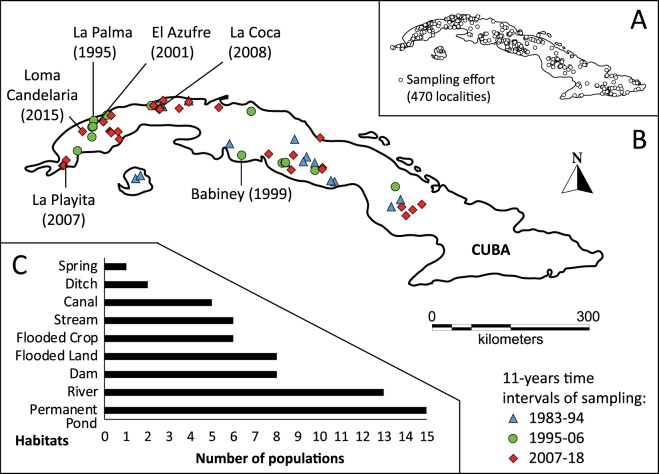
(**A**) Distribution of all registered records in the National Malacology Reference Database accounting for freshwater mollusc species from 1982–2018. (**B**) Updated distribution of *Pseudosuccinea columella* from Cuba. Positive sites were plotted with different points representing 11-year time intervals to account for historical and newly recorded data. Resistant populations are named in the map, consistently with previous published works. The year of first record of resistant populations is also indicated. (**C**) Number of records of *P*. *columella* per type of habitat. (Laboratory of Malacology, IPK).

Knowing the distribution of a snail species is particularly important to assess its epidemiological significance and to attempt its effective control. The National Reference Database contains nearly 500 records in Cuba (Fig. 2A), from which 68 verified sites harbour *P*. *columella* species (Fig. 2B). The distribution of *P*. *columella* ranges from the westernmost region (Pinar del Río province) to the east-central (Camagüey province) region of Cuba (Fig. 2B). No records exist for the easternmost provinces. Notably, 59% of sampled populations were documented in the last 15 years. Overall, earliest records are more abundant in the west-central region while populations from east-central Cuba correspond mostly to newer samplings. From the 68 verified *P*. *columella* populations, only six are acknowledged as resistant to *F*. *hepatica* infection (*i*.*e*. snails displaying the characteristic mantle pigmentation pattern described by Gutiérrez *et al*.^19^ and showing no record of parasite development following exposure to different *F*. *hepatica* isolates). Populations of resistant individuals are mostly recorded in the west (Pinar del Río – Havana provinces) with the exception of Babiney, in Cienfuegos (central Cuba; Fig. 2B).

Concerning habitat type preferences, permanent ponds, rivers and dams harbour 62.5% of all *P*. *columella* records, with up to six different habitats concentrating the remaining populations (Fig. 2C). We did not find a clear pattern of habitat types for resistant populations which were observed in permanent ponds (2), flooded lands (1), springs (1), ditches (1) and canals (1). Globally, the number of populations recorded in open or connected water systems (*e*.*g*. rivers, streams, canals) is fewer than those of closed habitats (*e*.*g*. dams, ponds, flooded lands; Fig. 2C).

### Population genetic structure of *P*. *columella* in Cuba

**Figure 3:**
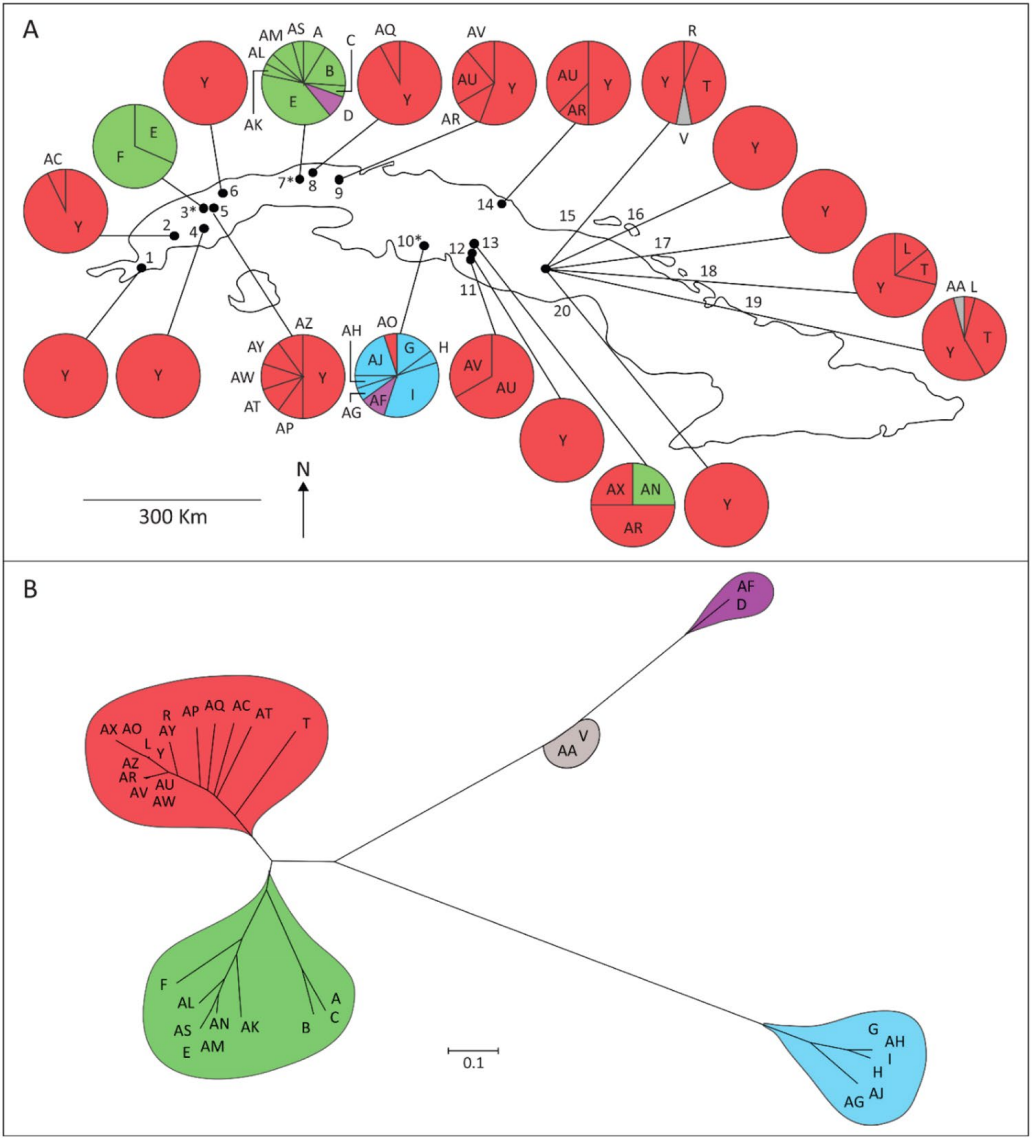
(**A**) Map showing the distribution of the different multilocus genotypes found (MLGT; one letter or letters’ combination = one MLGT = unique combination of alleles across all analysed loci) on sampled *Pseudosuccinea columella* populations genotyped through 6 microsatellite loci (3, 7, 10: *resistant *P*. *columella* populations). (**B**) MLGT of *P*. *columella* clustered following a joining-neighbour network using Nei’s genetic distance from each MLGT (different filling pattern correspond to different clusters). 1: Tio Pancho, 2: SJM, 3: El Azufre*, 4: Río Hondo, 5: IPA, 6: Pilón, 7: La Coca*, 8: Parque Lenin, 9: V7, 10: Babiney*, 11: Vega Grande, 12: El Antojo, 13: El Cacao, 14: Río Manaquita, 15: Río Yayabo, 16: Puesto de Mando, 17: Guillén, 18: Río Central, 19: Matadero Aves, 20: Arroyo.

Population genetic studies on *P*. *columella* could give insights into important questions for biological and health sciences; its evolutionary history in Cuba, its pattern of colonization, the genetic structure of its populations and its role in *F*. *hepatica* transmission. Thus, from the distribution of *P*. *columella* presented above, we selected 20 populations (see Fig. 3 for details) to analyse the genetic structure of this species in Cuba. Records of *P*. *columella* infected with different trematode species in Cuba exist in our laboratory but, in the present study, dissection of 329 snails only evidenced infection of *F*. *hepatica* in two individuals from IPA population (10.5% prevalence, IPA site). No other infections, by *F*. *hepatica* or other trematode species, were recorded at any other site.

The six microsatellite loci explored were polymorphic in *P*. *columella*, especially PCO12 which displays seven different alleles in Cuba. Population genetic parameters are presented in Table 1. Allelic richness is very low in the 20 studied populations (mean 2.034 ± 0.646 SD) and five populations (Pilón, El Antojo, Puesto de Mando, Guillén and Arroyo) are completely monomorphic at the six loci. The highest allelic richness was observed in the two resistant populations of La Coca and Babiney (2.17 ± 0.75 SD). However, the analysis of the third resistant population (El Azufre) showed a lower allelic richness (1.17 ± 0.41 SD). Expected heterozygosis (mean 0.1 ± 0.09 SD) and observed heterozygosis (0.03 ± 0.056 SD) were very low. Most populations had high and significant values of *F*_IS_ after Bonferroni correction (mean 0.667 ± 0.313 SD) for which estimated selfing rates were also high ( > 64%). Although an overall value of *F*_ST_ of 0.339 (*P* > 0.05) was observed, significant differentiation was only recorded in resistant populations (La Coca, Babiney and El Azufre) that not only differed from susceptible populations but also from each other (Supplementary Table S1).

**Table 1:** Mean allelic number (a), observed (*H*_o_) and expected (*H*_e_) heterozygosis, *F*_IS_ (level of significance after Bonferroni correction) and estimated auto-fecundity rates (*s*) for resistant (^R^) and susceptible (^S^) populations of *Pseudosuccinea columella* (N: number of analysed individuals).

Population	N	*a* (±DS)	H_o_ (±DS)	H_e_ (±DS)	*F*_IS_ (*P*)	*S*
^R^La Coca	37	2.17 ± 0.75	0.056 ± 0.074	0.304 ± 0.227	0.816 (<0.01)	0.9
^R^Babiney	26	2.17 ± 0.75	0.068 ± 0.059	0.249 ± 0.197	0.726 (<0.01)	0.84
^S^IPA	19	2 ± 1.1	0.068 ± 0.121	0.127 ± 0.164	0.469 (<0.01)	0.64
^S^Parque Lenin	26	1.67 ± 0.82	0.019 ± 0.045	0.087 ± 0.104	0.786 (<0.01)	0.88
^S^Río Arimao	13	1.83 ± 0.75	0.249 ± 0.259	0.239 ± 0.267	0.038 (NS)	0.08
^S^Río Hondo	15	1.5 ± 0.84	0.025 ± 0.039	0.092 ± 0.191	0.733 (<0.01)	0.85
^S^Río Manaquita	15	1.5 ± 0.84	0.028 ± 0.044	0.149 ± 0.25	0.814 (<0.01)	0.9
^S^Río Yayabo	20	1.33 ± 0.52	0.01 ± 0.025	0.174 ± 0.27	0.94 (<0.01)	0.97
^S^Tío Pancho	22	1.17 ± 0.41	0.008 ± 0.019	0.008 ± 0.02	0 (NS)	1
^S^V7	13	1.33 ± 0.52	0.031 ± 0.049	0.101 ± 0.212	0.689 (<0.05)	0.82
^S^Vegas Grandes	10	1.33 ± 0.52	0.05 ± 0.083	0.117 ± 0.24	0.571 (NS)	0.73
^S^Arroyo	16	1 ± 0	0	0	—	1
^S^Puesto de Mando	6	1 ± 0	0	0	—	1
^S^El Antojo	6	1 ± 0	0	0	—	1
^R^El Azufre	19	1.17 ± 0.41	0	0.076 ± 0.186	1 (<0.01)	1
^S^Guillén	15	1 ± 0	0	0	—	1
^S^Río Central	7	1.5 ± 0.84	0.048 ± 0.073	0.091 ± 0.163	0.478 (NS)	0.65
^S^Matadero de Aves	24	1.5 ± 0.84	0.007 ± 0.017	0.17± 0.263	0.959 (<0.01)	0.98
^S^Pilón	6	1 ± 0	0	0	—	1
^S^SJM	14	1.17 ± 0.41	0	0.024 ± 0.058	1 (NS)	1

Multilocus genotyping at the six microsatellite loci showed the existence of 36 MLGT. Observed mean number of MLGT in susceptible populations was 2.764 (±1.562 SD) while a higher average of 6.333 (±3.785 SD) was attained in resistant populations. In particular, differences in MLGT between each group of populations were statistically significant (t value (df: 18) = 3.58; *P* = 0.002). Mean genotypic diversity was 0.397 (±0.357) with the highest values in the resistant populations of La Coca (0.893) and Babiney (0.836). In the case of resistant individuals, 18 MLGT were observed for which only MLGT *E* is shared in two populations (El Azufre and La Coca). Figure 3A shows the distribution of the 36 MLGT in Cuba with the MLGT *Y* widespread in most sampled sites and the only one observed in susceptible monomorphic populations. This MLGT *Y* is absent in resistant populations (Fig. 3A). Interestingly, the two infected individuals found in the IPA population and the population from Pilón previously reported as infected (see Gutiérrez *et al*.^18^), shared the same MLGT *Y*. A different MLGT (*T*) was observed only in the easternmost populations (Río Manaquita, Río Central and Matadero Aves) within several individuals. The rest of the MLGT are considered rare and differ from one another by a single or few alleles. The obtained MLGT network from Nei’s genetic distance displays three majoritarian clusters (Fig. 3B). Susceptible MLGT gathered within one major cluster. Two different major clusters and one small cluster contain most of the MLGT from resistant individuals (Fig. 3B).

### Ecological patterns associated with the occurrence of resistant and susceptible *P*. *columella* in Cuba: field and laboratory observations

The higher allelic and MLGT richness of resistant populations presented herein contrasts with their restricted distribution, as they have been identified in only six of 68 sites harbouring *P*. *columella*. In this sense, studies depicting more general ecological interactions of both susceptible and resistant *P*. *columella* in the mollusc assemblage and their interaction with the abiotic factors, which could explain the differential distribution observed, are lacking. Considering that such resistance could hold promise as a control strategy to disengage *F*. *hepatica* transmission in the field, it is important to account for its ecological or demographical patterns. Thus, we analysed the occurrence of each phenotype in the wild in association with different environmental factors and we carried out experimental life tables to address this topic.

#### Reviewing data from natural populations

Since no apparent pattern concerning the type of habitat was found in either resistant or susceptible *P*. *columella* (see section 3.1), we aimed to identify specific ecological features associated with the occurrence of each phenotype. For this, we used data from 14 sites harbouring *P*. *columella* mostly concerning different abiotic factors (see Table 2). It can be noted, that both susceptible and resistant populations seem to prefer water with low nitrite and ammonium concentrations. Interestingly, the recorded ecological data showed a tendency for resistant populations to settle in waterbodies with lower pH (6–6.5), TH (4–9°d) and mollusc richness (see Table 2).

**Table 2:** Mollusc species richness (S), dissolved ions (P0_4_^3−^, NO_3_^−^, NO_2_^−^, NH_4_^+^), O_2_ and pH and total hardness (TH) from sampled sites harbouring resistant (^R^) and susceptible (^S^) *Pseudosuccinea columella*.

Population	S	P0_4_^3-^	NO_3_^−^	NO_2_^−^	NH_4_^+^	O_2_	pH	TH (°d)	Reference
^R^La Palma	3	—	50	0.1	0.2	—	6.5	6	This study
^R^El Azufre	7	0.5	10	0.05	0.2	—	6	9	^49^
^R^La Coca	3	0.25	—	0.05	0.2	15	6	4	This study
^R^La Playita	2	—	—	—	—	—	6.5	—	This study
^R^Candelaria	1	—	—	—	—	—	6	6	This study
^S^Negrines	5	0.5	—	0.05	0	20	7.5	20	This study
^S^Batabanó	11	—	—	—	—	—	8	20	^53^
^S^Botánico	7	—	0	0.1	0.4	—	8	11	^36^
^S^El Rubio	7	—	25	0.05	0.2	64	8	22	This study
^S^Parque Lenin	15	0.25	5	0.1	0	68	8	18	This study
^S^Bodegón	4	—	25	0	0	—	7.5	22	^36^
^S^San Juan Martinez	7	0.5	10	0.05	0.2	——	7.5	8	This study
^S^Camagüey	8	—	—	—	—	—	7	18	This study
^S^Hanabanilla	12	—	21	—	0.03	—	7.2	4	^54^
^R^Mean ± SE	3.2 ± 1.02	0.38 ± 0.13	30 ± 20.00	0.067 ± 0.01	0.2 ± 0.00	—	6.2 ± 0.12	6.3 ± 1.03	—
^S^Mean ± SE	8.4 ± 1.18	0.41 ± 0.08	14.33 ± 4.4	0.058 ± 0.01	0.12 ± 0.06	50.7± 15.37	7.6 ± 0.13	16.2 ± 2.19	—

Data from unpublished field notes archived in the Laboratory of Malacology, IPK, Cuba are referred to in this table as ‘this study’. SE: standard error.

#### Ecological factors affecting the abundance of resistant and susceptible *P*. *columella* in the La Coca locality

We found the two distinct phenotypes (resistant and susceptible) of *P*. *columella* occurring in the La Coca locality, but with a marked degree of ecological segregation depending on the particular sampled site, with Segundo Potrero only harbouring resistant snails (La Coca resistant population). *G. cubensis* (Lymnaeidae), *Physa acuta* (Physidae), *Drepanotrema anatinum* and *Drepanotrema lucidum* (Planorbidae) were recorded in Negrines. In this site, *P*. *acuta* and *D*. *anatinum* showed similar variations while *G*. *cubensis* performed two ephemeral colonisations with rapid extinctions (Supplementary Fig. 1). *Physa acuta* was dominant in Negrines but it did not seem to affect the population dynamic of *P*. *columella* (Supplementary Fig. 1). In contrast, the scenario at Segundo Potrero was completely different with a very low snail species richness marked by low abundances of *D*. *anatinum* and *Gundlachia radiata*, and a considerable dominance by resistant *P*. *columella* snails (Supplementary Fig. 1).

**Figure 4:**
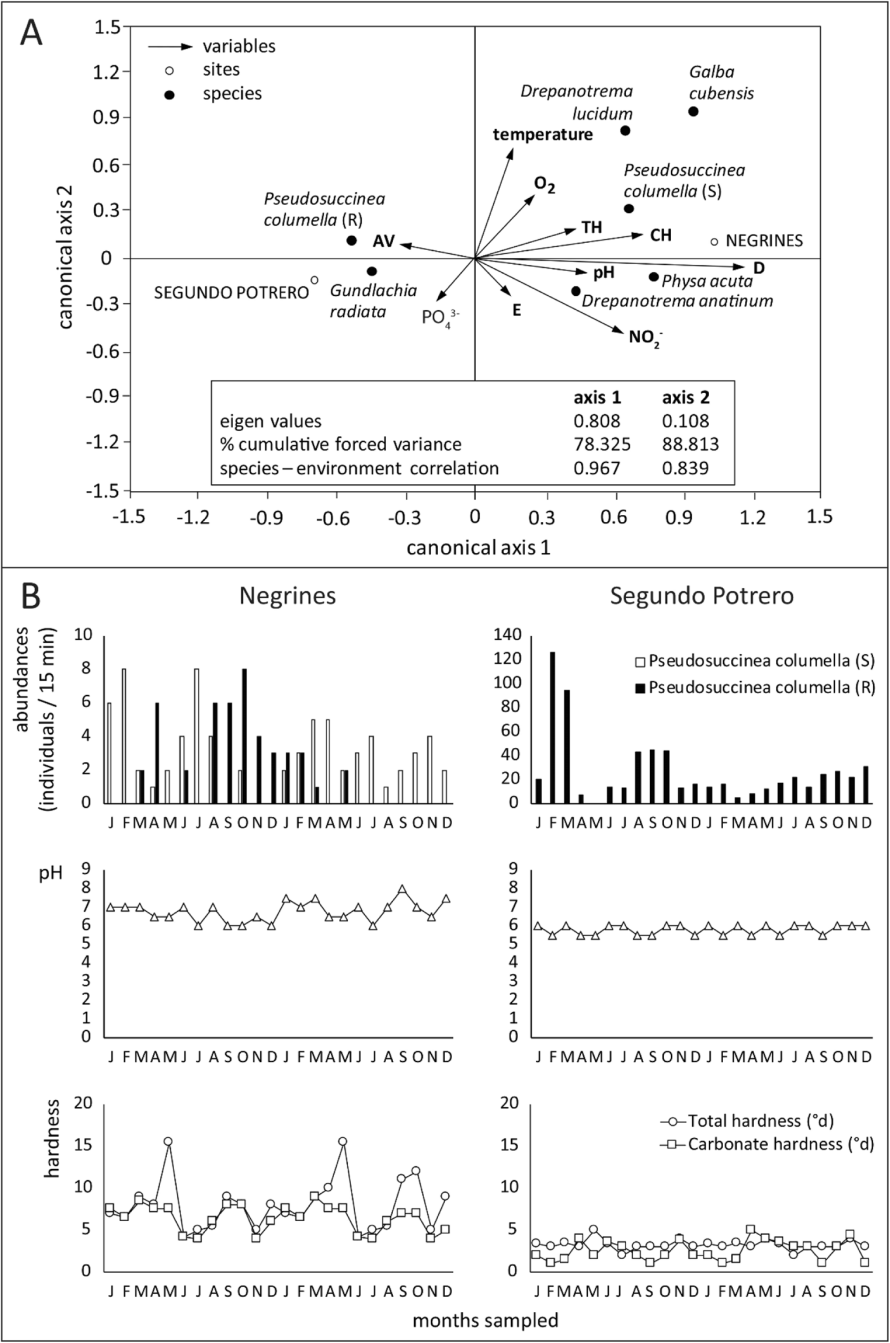
(**A**) Canonical correspondence analysis of the two-year follow up study in the La Coca locality (sampled sites: Negrines and Segundo Potrero). (**B**) Monthly relative abundances of *Pseudosuccinea columella* and dynamics of pH and water hardness in the two sampled sites within the La Coca locality. AV: aquatic vegetation, CH: Carbonate hardness, D: Simpson’s Diversity index, S: susceptible *P*. *columella*, R: resistant *P*. *columella*, T: temperature, TH: Total hardness.

The ecological follow-up was only carried out in two sites, which limits replication. However, the closeness and constant water flow interchange between both sites and the ecological variations recorded on some variables in the Negrines site allowed us to observe an interesting pattern. Concerning the different phenotypes of *P*. *columella*, we should note that, while in Segundo Potrero only resistant individuals are found (*i*.*e*. La Coca population), in Negrines susceptible individuals occurred in sympatry with resistant snails at certain times of the study (Fig. 4B). The canonical correspondence analysis performed with all the ecological data recorded at both sites showed a particular relationship between the abundances of resistant and susceptible individuals with variations in pH and both TH and CH (Fig. 4A). We found a strong negative association between resistant snails and pH increase demonstrating a certain tolerance for slightly acidic waters. The remaining factors had a minor effect on species abundances. In this sense, while in Negrines the mean values of pH and TH were around 7.5 and 14°d respectively, Segundo Potrero showed lower values (pH: 5.5–6; TH: 2°d-4°d) with an annual stability and a high abundance of resistant *P*. *columella*. However, in Negrines, the population of *P*. *columella* remained stable at low effective size but with peaks of resistant individuals when susceptible snails decreased or disappeared. Interestingly, such colonization events by the resistant snails in Negrines matched a drop in pH and TH values highlighting their particular association with water acidity and TH observed in the canonical analysis (Fig. 4) and with the overall pattern recorded from the previous analysis (section 3.3.1). Globally, susceptible snails were more abundant when pH ranged between 7 and 8.

Notably, we failed to detect *F*. *hepatica* infection from *P*. *columella* during the two-year ecological follow-up study in La Coca. However, while susceptible snails from Negrines were experimentally infected, it was not possible to infect resistant snails with different *F*. *hepatica* isolates.

#### Influence of pH and total hardness on experimental life history traits of resistant and susceptible *P*. *columella*

Given that the evidences gathered from the historical field notes and from the population dynamics in the La Coca locality suggested a differential relation between the occurrence of susceptible and resistant individuals concerning the pH/TH (*i*.*e*. positive association of resistant populations with acidic and soft waters with low species richness), we performed an experimental life table study to assess the effect of these abiotic factors on several life-history traits of *P*. *columella*. We measured survival and reproductive traits as shell size showed no significant differences between *P*. *columella* phenotypes in a previous study^24^. Age-independent parameters, and life expectancy and fecundity rates for weeks 0, 5, 9 and 14; considering the start of the experiment, sexual maturity, middle and late snail developmental stages respectively, are shown in Table 3. To facilitate comprehension and to elucidate patterns, we will address the results from two different levels of analysis: (a) the overall effects on the species and (b) the differential effects on the resistant/susceptible phenotypes.

**Table 3:** Experimental life-history traits of resistant (R: La Palma and La Coca) and susceptible (S: Aurora and Negrines) *Pseudosuccinea columella* at different pH and total hardness (TH) conditions (e_x_: life expectancy, m_x_: fecundity rate, NHS within one week: snails that hatched within one week after egg laying, Ro: net reproductive rate, FR: week at first reproduction, *r*: intrinsic rate of natural increase, λ: finite rate of natural increase.

Condition(pH/TH)	Strain	Age-dependent traits							Age-independent traits				NHS within one week (%)	Viable eggs (%)
		W0	W5		W9		W14							
		e_x_	e_x_	m_x_	e_x_	m_x_	e_x_	m_x_	FR	Ro	r	λ		
5.9/4°d	La Palma^R^	10.9	5.9	0.7	3.7	12.7	2.3	115.5	4	37.4	0.72	2.06	72.9	99.37
	La Coca^R^	10.3	5.3	0.6	5.5	27.8	6.1	96.7	5	116.8	0.79	2.20	68.4*	96.46
	Aurora^S^	7.9	2.9	1.5	5.0	76.5	1.5	147.0	4	37.8	0.73	2.06	53.9*	95.4*
	Negrines^S^	4.4	4.4	25.0	3.3	28.0	—	—	4	19.2	0.59	1.80	64.8*	67.5*
7.6/14°d	La Palma^R^	8.6	6.7	1.1	4.0	5.3	2.2	56	3	45.1	0.95	2.59	71.5	98.3
	La Coca^R^	17.4	13.8	0.2	9.8	0.7	5.0	10.6	4	56.9	0.81	2.24	97.4*	98.7
	Aurora^S^	12.1	7.6	0.8	5.0	2.6	2.7	33.4	3	56.4	1.00	2.74	92.5*	96.6*
	Negrines^S^	12.7	8.9	0.3	5.2	0.3	2.5	20.4	4	21.8	0.62	1.85	87.6*	99.2*

Data corresponds to weeks (W) 0, 5, 9 and 14. The (*) represents significant differences (*P* <0.05) for each population when comparing the given trait between experimental settings by Fisher’s Exact Tests.

**Figure 5:**
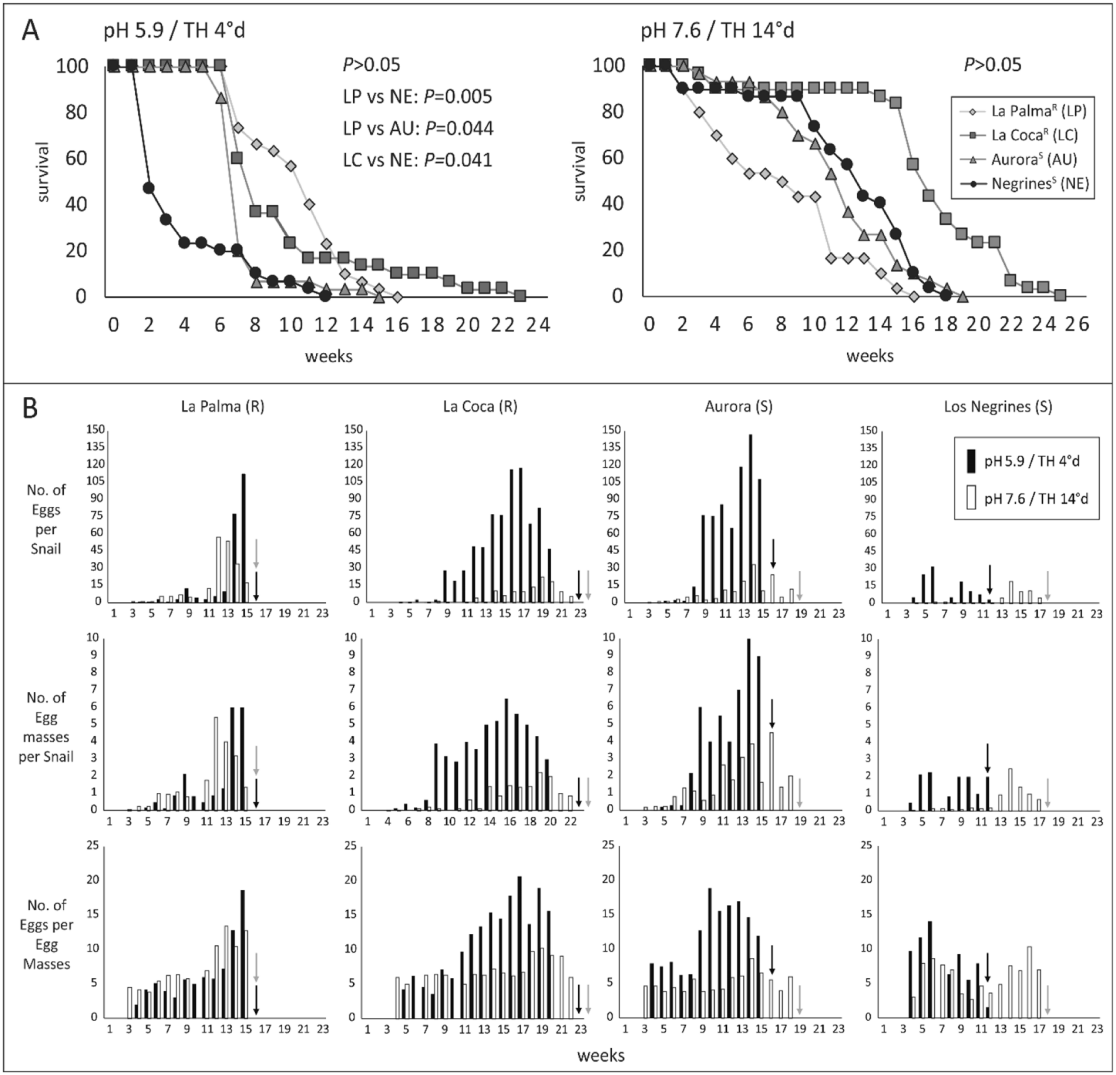
(**A**) Survival curves of resistant (R) and susceptible (S) *Pseudosuccinea columella* populations in which pairwise comparisons of snail survival between population at each experimental setting, performed by log-rank Tests, are also shown. (**B**) Egg production data of experimentally reared *P*. *columella* snails at pH and total hardness (pH/TH) of 5.9/5°d or 7.6/14°d. Arrows within the egg production data indicate the week at which 100% mortality was observed for the given population at each condition. Results of Factorial ANOVAs were *P* < 0.05.

*Overall life-history traits for Pseudosuccinea columella – common pH/TH conditions seem better suited to P*. *columella species*: Survival of *P*. *columella* was highest during the first eight weeks with a lifespan ranging from 15 to 23 weeks (3 to 5 months). Sexual maturity was reached between the third and fifth weeks, depending on the population and experimental setting. Reproductive traits showed a steady increase since first egg laying until reaching a peak between week 13 and week 16 (see Table 3 and Fig. 5 for details).

Overall, *P*. *columella* performed better at common pH/TH conditions irrespective of the phenotype (see Table 3; Fig. 5). Low pH/TH values had a negative impact on snail survival with the lowest life expectancies compared to common conditions (Table 3). In particular, with the exception of La Palma, the other populations considerably decreased their life expectancy at birth (La Coca: 1.7-fold, Aurora: 1.5-fold, Negrines: 2.9-fold). Regarding mortality, this trait peaked at week 1 for Negrines and around week 7 for the remaining populations (*i*.*e*. all populations but La Palma were reduced by more than half; Fig. 5A, Table 3). Furthermore, fertility traits were also negatively affected under these conditions. A significant delay in snail hatching within the first week following egg laying was recorded at low pH/TH for all populations but La Palma (NHS within the first week in Table 3). Additionally, several egg masses laid and incubated at 5.9/4°d needed up to three weeks to hatch. The combination of these later effects also affected population increases (r and λ) which were lower at low pH/TH. Net reproductive rate was also lower at 5.9/4°d except for La Coca which presented a two-fold higher Ro (Table 3). All these impairments on survival and reproductive parameters of *P*. *columella* reared at low pH/TH strongly suggests that common conditions of 7.6/14°d are better suited to this species, irrespective of the phenotype.

Interestingly, overall higher fecundity rate was observed at 5.9/4°d (Table 3) probably to compensate for the reduction in self-maintenance and reproduction recorded on *P*. *columella* under these conditions. However, a significant increase of the mean number of eggs, egg masses and egg per egg masses was only attained by La Coca and Aurora snails at low pH/TH (Fig. 5B; Tukey’s Test post hoc, *P* < 0.05).

*Differential effects of pH and TH at the resistant/susceptible phenotype level – higher tolerance to low pH/TH conditions is possibly associated with the resistant phenotype:* Despite the overall decrease in self-maintenance and reproduction of *P*. *columella* at 5.9/4°d presented above, differences can be seen between phenotypes that points at a higher tolerance for low pH/TH of resistant versus susceptible populations. In general, higher survival (Log rank Tests, *P* < 0.05; Fig. 5A) and life expectancy at birth (Table 3) were observed in resistant snails at low pH/TH conditions. In addition, we recorded a higher percentage of viable eggs in resistant compared to susceptible populations at low pH/TH (Table 3; Fisher’s Exact Test, *P* < 0.05). No significant variation in this trait in resistant *P*. *columella* at common versus low pH/TH (Table 3; Fisher’s Exact Tests, *P* > 0.05) was recorded. Conversely, an overall significant decrease in the proportion of viable eggs was observed on susceptible populations at 5.9/4°d compared to 7.6/14°d (Table 3; Fisher’s Exact Test, *P* <0.05).

*Differences in life traits between populations (irrespective of the experimental setting) with focus on reproductive outputs:* It is worth mentioning that different patterns regarding reproductive traits were recorded for each population independent of the pH/TH conditions. In susceptible *P*. *columella*, the Aurora isolate was characterized by high values of Ro, r and λ (Fig. 5; Table 3) while a more discrete output of laid eggs per snail was recorded in Negrines for both conditions (Fig. 5B for details). Interestingly, the resistant La Coca population presented an overall lower fecundity rate than both susceptible populations (see Table 3). In addition, egg masses/snail (attempts to reproduce) in this population always peaked the latest (weeks 16–17 at low pH/TH and 19–20 at common pH/TH) with the exception of Negrines at low pH/TH that instead of  a marked peak, showed a relatively steady production since early stages. In the case of resistant La Palma we observed that, regardless of the experimental condition, around 30% of their eggs needed more than one week to hatch (see NHS in Table 3). Even at 7.6/14°d, where the reproductive output of this population was higher (high values of Ro, r and λ), this delay in egg hatching remained unaltered (Fisher’s Exact Test, P > 0.05; Table 3). Additionally, even after the increase of Ro, r and λ observed at 7.6/14°d (see Table 3), the age-independent reproductive traits of La Palma, although outperforming Negrines, were lower to the susceptible population of Aurora.

## Discussion

### Distribution and population genetics of *P*. *columella* in Cuba: insights into its history and colonization and its relationship with *Fasciola hepatica* transmission

*Pseudosuccinea columella* is native to North America but has been widely introduced outside of its native range due to natural (*e*.*g*. aquatic birds, flooding) or human-mediated invasion^7^. The date of introduction of *P*. *columella* in Cuba is unknown, but it was first reported in 1858 by Poey under the name of *Lymnaea francisca*. It is thus plausible that a first introduction in the beginning of the 19^th^ century allowed *P*. *columella* to settle in the western part of Cuba, the region where the oldest and abundant known documentations have been described. The close proximity of Cuba to the North American continent provides an easy opportunity for natural introductions, particularly in the westernmost region. Our results reveal several facts suggesting that successive introductions of *P*. *columella* occurred successfully in Cuba, mainly in the west but also in the central region. A sluggish invasion of this species to the eastern region of the island, mostly by more recently introduced invasive genotypes (*e*.*g*. MLGT *Y*), is presumed to be ongoing. Perhaps the most obvious evidences to support this assumption are: (1) the increased number of records in recent years from the east-central region and its complete absence in the easternmost region; (2) the higher allelic and multilocus genotypic richness in the west (particularly in resistant populations); (3) the existence of three major clusters of MLGTs and the presence of some MLGTs only found in the central region of Cuba, which contrasts with (4) the similar number of MLGTs shared by most susceptible *P*. *columella* populations (with five that were completely monomorphic) and the widespread distribution of MLGT (*Y*) in Cuba. The overall low genetic structuration and diversity found in *P*. *columella* can be explained by recent genetic bottlenecks that usually occur after introduction events, followed by the expansion of certain invasive genotypes such as MLGT *Y* (see Lounnas *et al*.^7^).

In particular, previous studies showed that resistant *P*. *columella* populations differ from those which are susceptible. Gutiérrez *et al*.^23^ detected different RAPD profiles using 17 different primers. Another study by Calienes *et al*.^21^ was the first to attempt to detect clear segregation when comparing polymorphic DNA of three resistant populations (La Palma, El Azufre, Babiney) and nine susceptible populations from Cuba using RAPD profiles. In the present study, we found that the genetic structure of the three resistant populations analysed (El Azufre, La Coca and Babiney) is significantly different in terms of presence and amount of MLGTs from susceptible populations, with higher genotypic diversity. The clear segregation into different susceptible and resistant MLGT associated clusters observed here, support the idea of an early, different and detached history from susceptible *P*. *columella* populations. In contrast to highly susceptible populations, resistant MLGTs from western and central populations cluster separately from each other, and it is thus likely that they originated after different introduction events. However, we should note that we have only tested three out of the six known resistant populations, and thus the three others remain unexplored.

Concerning parasite transmission, the observed distribution pattern (range and number of populations) in *P*. *columella* compared to the other lymnaeid *G*. *cubensis* (over 100 recorded populations all over the Cuban archipelago; see^15^) supports the presumed secondary role for this species as an intermediate host of *F*. *hepatica* in Cuba. A recent global-scale genetic study on *P*. *columella* considered one of our recorded MLGT (*T*) as highly invasive after it was detected in Colombia, Peru, Venezuela, South Africa and the Indian Ocean^7^. Surprisingly we only found this MLGT in two eastern populations in Cuba (Río Central and Matadero Aves) suggesting that it may be a recent introduction and may pose a risk of further spread in Cuba. Additionally, MLGT *Y* which is only found in monomorphic populations, is most likely the result of a recent introduction and may in fact present itself as ecologically adaptable (found in 15 out of 20 analysed populations) and highly compatible with *F*. *hepatica*. For instance, the five individuals found naturally-infected with *F*. *hepatica* in Cuba correspond to this specific MLGT (two individuals from IPA and three from Pilón). From this we could expect that if populations with a tendency to become infected matched those that present a high ecological plasticity, a highly favourable scenario for *F*. *hepatica* transmission in Cuba would result, since it would increase the probability for the parasite to find a suitable host. However, this hypothesis would benefit from the identification of more infected individuals that share the same GTML. In any case, the lack of genetic diversity has already been recognised as an advantage for parasites to proliferate in a given host population^41^, and overall, susceptible populations of *P*. *columella* showed no significant differentiation (low F_ST_, *P* > 0.05).

In the present study, the low allelic richness per population as well as the strong deviations from panmixia suggest a high self-fertilization rate in this species, as also demonstrated by Lounnas *et al*.^7^. Other lymnaeid snails such as *G*. *truncatula*^42^ and *Omphiscola glabra*^43^ also prefer self-fertilization as a reproductive mode. Similarly, other studies observed the same low allelic richness pattern in *P*. *columella* that we found^27^. However, here, three populations of *P*. *columella* (IPA, Río Central and Río Arimao) showed lower self-fertilization rates than those previously reported. Several studies indicated that cross-fertilization may represent a selective advantage when populations are under severe parasitic pressures^44,45^. Conversely, self-fertilization can be selected in stochastic environments^46^ securing reproduction even if only one individual survives the harsh conditions. Thus, we should expect lower self-fertilization rates in stable habitats or endemic areas for parasite transmission. The IPA site matches both criteria since it was previously suggested as a fasciolosis transmission site by Gutiérrez *et al*.^47^, and consists of a permanent pond used to stock water. In fact, the two infected individuals belonged to this population. The other two sites (Rio Central and Rio Arimao) are recognised as fasciolosis areas as high bovine prevalence are reported^48^.

While the establishment of resistant populations decreases the chances of transmission of the liver fluke, the observed distribution pattern of such phenotype even with a high genetic diversity and an older presence of the resistant population in Cuba suggests that there is an ecological cost for resistance. The latter was previously proposed by Gutiérrez *et al*. after performing experimental history traits following *F*. *hepatica* infection^24^ and under competition^25^. Thus, a deeper look into the ecological patterns associated with the occurrence of this phenotype in nature is necessary in the pursuit of using this phenomenon to develop novel control strategies for *F*. *hepatica* transmission.

### Ecological patterns associated with resistant and susceptible *P*. *columella* from Cuba: insights into the cost of resistance

Lymnaeid snails are known to occur worldwide in an extensive range of habitat types^1^. In Cuba, it has been previously shown that *P*. *columella* does not exhibit particular preferences regarding natural or transformed habitats and contrasts with its relative *G*. *cubensis*, commonly established in anthropic sites^15^. *Pseudosuccinea columella* is highly aquatic and consequently could be more severely affected by the physical and chemical factors of the water, a fact that could be linked to its overall preference for closed freshwater systems and, in particular, to habitats like permanent ponds as recorded herein. A previous study by Gutiérrez^49^ in Pinar del Río, Cuba, showed a positive correlation between the abundances of *P*. *columella*, the pH and the concentrations of nitrites. Here, we reveal a different effect when the overall pattern of occurrence of resistant and susceptible individuals and the ecological dynamics of those living in sympatry, are considered separately. As presented here, the conditions for occurrence of susceptible individuals in terms of pH, TH and the concentration of nitrites and phosphates are similar to those reported by Perera^36^ elsewhere in Cuba. However, resistant snails present a marked association to lower values of water pH and TH and a tendency to colonize sites with low species richness. This result could be explained by two hypotheses:

(1) One hypothesis could be that these conditions are essential for the survival of resistant snails in the field (*i*.*e*. resistant snails could not tolerate other conditions), suggesting that they have a different ecology than susceptible snails. Such marked ecological segregation might lead us to think in a strong differentiation between phenotypes that, over time, would result in speciation. However, we have successfully kept resistant *P*. *columella* in our laboratory for more than 20 years at pH/TH conditions ranging between 7–8/12–18°d. In addition, invasive *F*. *hepatica*-susceptible *P*. *columella* populations have been reported in France at sites characterized by acidic soils and soft waters^50,51^. Both facts suggest that, as a single species, both *P*. *columella* phenotypes can tolerate pH and TH variations rather than be differentially-restricted to specific values of these parameters. Our experimental life-history traits also support that since *P*. columella, regardless of the phenotype, can survive within the same range of pH/TH conditions (5.9–7.6/4–12°d). However, while the species seems to be better suited to 7.6/12°d conditions, an overall higher performance (*i*.*e*. higher tolerance) at low pH/TH conditions, was observed for resistant individuals compared to susceptible populations, in terms of self-maintenance (survival and life expectancy) and reproductive (egg viability) traits.

(2) Thus, a second hypothesis could be that low pH and TH are much less suitable for other species (with narrower tolerance levels) and that these low values directly reduce competition. In this sense, our results demonstrate that such conditions negatively affect some demographic traits of *P*. *columella* in favour of resistant phenotypes. It is therefore difficult to provide an explanation for how resistant populations are maintained in ‘neutral’ (where common pH/TH levels are present) sites unless colonisation events by competitor snails are rare and that resistant individuals were the only ones to settle. This scenario was observed within our field results since the colonization of Negrines by resistant snails coincided with a decrease of pH and TH, and lower abundances of susceptible individuals (Fig. 4). This hypothesis can also be supported by the observed pattern of low richness of snail species in slightly acidic/soft water-sites where resistant populations are found. In a colonisation event of suitable sites by susceptible *P*. *columella* a replacement of the resistant snails might be expected. In fact, under experimental conditions, Gutiérrez *et al*.^25^ reported that resistant *P*. *columella* are less competitive than susceptible snails in terms of reproductive traits (reduced Ro when raised in the presence of susceptible snails). The latter contrasted with an increase in shell growth and Ro on susceptible *P*. *columella* when reared in competition with resistant individuals^25^. To summarise, the restricted distribution and the overall negative correlation of resistant *P*. *columella* and species diversity may be interpreted as an ecological cost of resistance, explained by a less competitive potential of resistant snails.

Assessing the competitive potential of resistant isolates in relation to susceptible *P*. *columella* and other freshwater snail species could be experimentally challenging and a demonstration of a definitive cost of resistance will require rearing in competition with one another, both *P*. *columella* phenotypes in the presence and absence of the parasite. However, the present study takes an important step forward in advancing our understanding of the suggested cost of resistance of *P*. *columella* from previous laboratory studies^24,25^, by recording a natural pattern and integrating field data and new experimental results into a plausible hypothesis. In this sense, while we cannot assure that susceptible populations outperform resistant isolates, evidence of possible trade-offs against reproduction in resistant populations were also found here (La Coca: lower fecundity rates compared to both susceptible isolates, late reproductive peaks); La Palma: delay of egg hatching regardless of the pH/TH condition, lower Ro compared to Aurora snails). Trade-offs are expected to occur from selecting advantageous features (*e*.*g*. resistance to parasite, ecological tolerance) and thus contribute to their low occurrence in the wild. In this sense, a previous study similarly observed reproductive constraints for La Palma (resistant) snails compared to other susceptible *P*. *columella* populations, particularly in terms of lower Ro^24^. Even considering the discrete performance of Negrines, the limited reproductive output whether in quantity (low fecundity rates) or in time (late peak of egg production or delay of egg hatching) observed here in resistant isolates, may negatively affect their success in colonizing new and highly suitable habitats. While several factors could account for the preclude of a natural dispersion of this phenotype in Cuba where the most common conditions of Cuban freshwater bodies include pH and TH ranging from 7–8 and 12–18°d^36^, identifying potential trade-offs associated with this phenotype might also bring forward some understanding of all possible outcomes of the suggested cost of resistance.

## Concluding Remarks

The introductions of vectors and intermediate hosts are significant issues because of the epidemiological risks they pose. In fact, recently introduced populations suffer strong genetic bottlenecks that drag off former genetic diversity preventing them from acquiring resistance to local parasites^41^. Genetic flow could act as a force that increases this diversity but, in contrast to what may be occurring in the liver fluke populations from Cuba (high cross-fertilization and bad management of cattle that mixes the strains;^52^), it is unlikely to take place at effective rates in *P*. *columella* snails. However, as discussed above, populations carrying some highly invasive MLGTs are colonizing a number of available sites in Cuba, one of which (MLGT *Y*) was also directly related to *F*. *hepatica* transmission in the present study. Thus, it is plausible that the ability of *P*. *columella* individuals with this MLGT to invade represents a true danger regarding transmission of the liver fluke. In any case, it is of major importance to maintain an active surveillance of parasite prevalence in *P*. *columella* and of potentially new introductions of this species in different and distant regions of Cuba and the world.

On the other hand, while the spread of resistant *P*. *columella* could definitively aid in the effective control of *F*. *hepatica* transmission, our results suggest that a resistance-related cost exists, as previously discussed by Gutiérrez *et al*.^24,25^. It is, thus, highly improbable that genotypes with invading abilities also correspond to those resistant to *F*. *hepatica*. The present work is a step forward towards the rational application of *P*. *columella* resistance as a potential variant to tackle parasite transmission. Our results provide insights into the ecological cost of resistance and the patterns associated with its occurrence in nature that could be used for planning a human-mediated introduction of resistant snails in particular high-risk transmission foci. Other strategies to incorporate resistant features into susceptible populations by out-crossing could be challenging if the high self-fertilization rates of *P*. *columella* showed herein and elsewhere^7^ are considered. On the other hand, other approaches like genetic manipulation, the controlled selection of resistant features or induction of resistance-mediated mechanisms in wild vector populations might turn out to be feasible. However, for any of these approaches to be applied, a deeper and wider comprehension of the molecular and immunological bases that mediate the natural resistance of *P*. *columella* to *F*. *hepatica* is required.

## Supplementary information

Supplementary information
