# Supplementary material for: Patterns of distribution, population genetics and ecological requirements of field-occurring resistant and susceptible Pseudosuccinea columella snails to Fasciola hepatica in Cuba
Source: Sci Rep. 2019 Oct 7;9:14359. doi: 10.1038/s41598-019-50894-7 (PMC6779948; doi:10.1038/s41598-019-50894-7)
Supplement: Supplementary file 1 — Supplementary information [file 41598_2019_50894_MOESM1_ESM.pdf]

Patterns of distribution, population genetics and ecological requirements of field-occurring resistant and susceptible *Pseudosuccinea columella* snails to *Fasciola hepatica* in Cuba

Annia Alba[a,b,1], Antonio A. Vázquez[a,c,1], Jorge Sánchez[a], Manon Lounnas[c], Jean-Pierre Pointier[d], Sylvie Hurtrez-Boussès[c,e], Benjamin Gourbal[b]

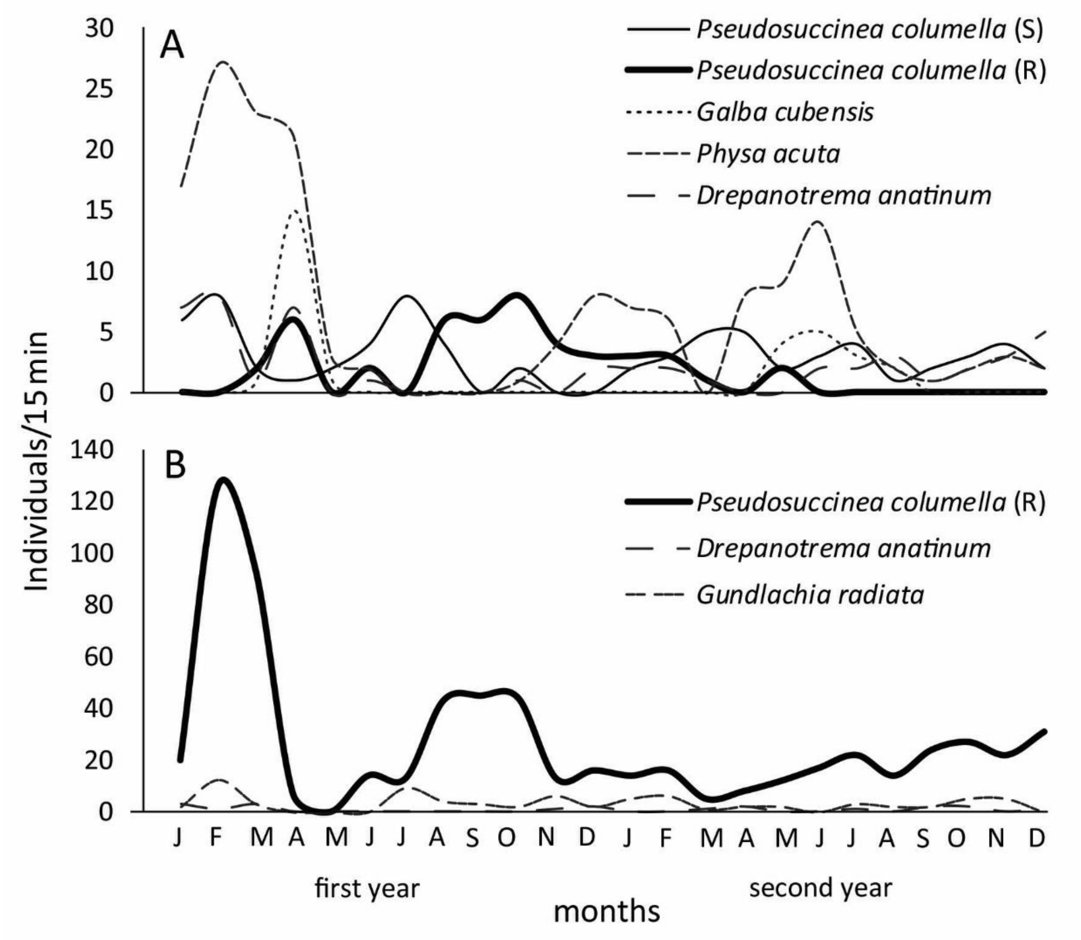

Supplementary figure 1. Monthly relative abundances of the freshwater mollusc species in the La Coca locality where resistant (R) and susceptible (S) *Pseudosuccinea columella* populations live in sympatry. Two sites were sampled: A) Negrines. B) Segundo Potrero.

Supplementary table S1.

Pairwise F_{ST} values between populations of *Pseudosuccinea columella* in Cuba **(populations: LC = La Coca, BA = Babiney, PL = Parque Lenin, RA = Río Arimao, RH = Río Hondo, RM = Río Manaquita, RY = Río Yayabo, TP = Tío Pancho, V7 = Vaquería 7, VG = Vegas Grandes, AR = Arroyo, PM = Puesto de Mando, AN = El Antojo, AZ = El Azufre, GB = Guillén y Boca, RC = Río Central, MA = Matadero Aves, PI = Pilón, SJM = San Juan y Martínez).**

	**LC**	**BA**	**IPA**	**PL**	**RA**	**RH**	**RM**	**RY**	**TP**	**V7**	**VG**	**AR**	**PM**	**AN**	**AZ**	**GB**	**RC**	**MA**	**PI**	**SJM**
**LC**	0,000																			
**BA**	**0,452***	0,000																		
**IPA**	**0,548***	**0,694***	0,000																	
**PL**	**0,598***	**0,730***	0,001	0,000																
**RA**	**0,321***	0,564	0,187	0,301	0,000															
**RH**	0,560	0,693	0,045	0,009	0,272	0,000														
**RM**	**0,501***	**0,664***	0,108	0,222	0,095	0,221	0,000													
**RY**	**0,548***	**0,697***	0,248	0,338	0,289	0,307	0,294	0,000												
**TP**	**0,616***	**0,748***	0,010	-0,023	0,344	0,149	0,271	0,353	0,000											
**V7**	**0,524***	**0,686***	0,020	0,120	0,141	0,168	-0,039	0,283	0,167	0,000										
**VG**	0,497	0,664	0,072	0,209	0,105	0,250	-0,107	0,272	0,314	-0,116	0,000									
**AR**	**0,623***	**0,771***	0,090	0,033	0,460	0,161	0,432	0,440	-0,007	0,386	0,587	0,000								
**PM**	**0,552***	**0,709***	0,006	-0,037	0,310	0,054	0,281	0,318	-0,041	0,224	0,385	NA	0,000							
**AN**	**0,552***	**0,709***	0,006	-0,037	0,310	0,054	0,281	0,318	-0,041	0,224	0,385	NA	NA	0,000						
**AZ**	0,301	**0,590***	**0,856***	**0,871***	**0,742***	0,862	**0,842***	**0,823***	**0,934***	**0,872***	**0,873**	**0,932***	**0,905***	**0,905***	0,000					
**GB**	**0,617***	**0,766***	0,085	0,029	0,449	0,153	0,421	0,431	-0,008	0,374	0,573	NA	NA	NA	**0,930***	0,000				
**RC**	0,521*	**0,685***	-0,036	-0,004	0,203	0,046	0,163	0,152	-0,017	0,084	0,154	0,154	0,022	0,022	**0,870***	0,144	0,000			
**MA**	**0,560***	**0,703***	**0,180***	0,253	**0,280***	0,234	**0,266***	-0,028	0,250	**0,230***	**0,235***	0,320	0,220	0,220	**0,812***	0,313	0,082	0,000		
**PI**	**0,552***	**0,709***	0,006	-0,037	0,310	0,054	0,281	0,318	-0,041	0,224	0,385	NA	NA	NA	**0,905***	NA	0,022	0,220	0,000	
**SJM**	**0,597***	**0,748***	0,071	0,028	0,385	0,111	0,362	0,396	0,017	0,295	0,448	0,010	-0,075	-0,075	**0,912***	0,005	0,074	0,291	-0,08	0,000

* bold values mean significant differentiation after the Bonferroni correction (adjusted $P = 0{,}000263$)
